# Use of Poly(styrene-*co*-acrylic Acid) in a Composite Ion-Solvating Membrane for Water Electrolysis

**DOI:** 10.1021/acspolymersau.5c00126

**Published:** 2025-12-19

**Authors:** Domenico Lentini, Francesko Malaj, Alessandro Tampucci, Lorenzo Brogi, Tommaso Caielli, Piercarlo Mustarelli, Pierpaolo Minei, Massimo Melchiorre, Oreste Tarallo, Francesco Ruffo

**Affiliations:** † Department of Chemical Sciences, University of Naples Federico II, Complesso Universitario di Monte S. Angelo, Via Cintia, Naples IT-80126, Italy; ‡ Ne.m.e.sys. s.r.l., via 2 Giugno 8, Sesto Fiorentino (Fi) 50019, Italy; § Department of Materials Science, 9305 University of Milano-Bicocca, Via Roberto Cozzi 55, Building U5, Milano 20125, Italy; ∥ SPIN-PET, Via R Piaggio, 32, Pontedera 56025, Italy; ⊥ Interuniversity Consortium for Chemical Reactivity and Catalysis CIRCC, Via Celso Ulpiani 27, Bari 70126, Italy

**Keywords:** hydrogen, ion solvating membrane, alkaline water electrolysis, poly(styrene-*co*-acrylic acid), composite membrane

## Abstract

Ion-solvating membranes (ISMs) have recently emerged as a promising class of materials for alkaline water electrolysis. Their non microporous polymer architecture and the absence of alkaline labile functional groups pave the way for a new generation of hybrid electrolyzers that combine the key features and advantages of conventional alkaline water electrolyzers (AWE) with those of proton exchange membrane water electrolyzers (PEMWE). Herein, a styrene-acrylic acid copolymer was synthesized and deposited onto a commercial microporous polypropylene support, yielding a composite ISM that couples an ion exchange capacity (IEC) of 2.72 mmol g^–1^ with good mechanical properties (tensile strength of 65 MPa). The electrochemical performance of the membranes was evaluated by through-plane conductivity measurements (24 mS cm^–1^, with KOH 4 M at 70 °C) and operation in an electrolyzer cell (reaching a cell voltage of 1.89 V at a current density of 1 A cm^–2^, with KOH 4 M at 70 °C). Polymer stability was assessed by monitoring structural changes via ^1^H NMR spectroscopy after an aging treatment in KOH 4 M at 70 °C for 720 h. Additionally, long-term electrolysis was investigated through a discontinuous cell test over 150 h. No detectable signs of degradation were observed in either test.

## Graphical abstract

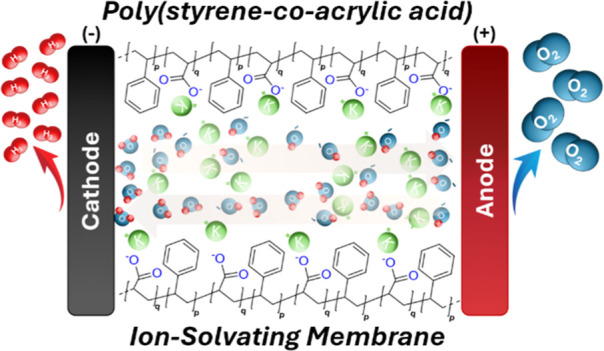

## 1 Introduction

Hydrogen is a key chemical widely employed in industry for ammonia synthesis, petroleum refining, and metallurgical processes.^1^ In recent years, increasing attention has been devoted to its potential use as an energy carrier and fuel.^1–3^ Currently, hydrogen is predominantly produced via methane steam reforming, which has a significant environmental footprint. In 2022, hydrogen production and use were associated with over 900 million tonnes of CO_2_ emissions.^1^ Water electrolysis offers a promising alternative for hydrogen production, particularly when performed using systems that can be efficiently coupled with electricity derived from renewable energy sources.^1^ The two most established and commercially available technologies for water electrolysis are proton exchange membrane water electrolyzers (PEMWEs) and alkaline water electrolyzers (AWEs).^4,5^ PEMWEs are known for their ability to operate at high current densities and with elevated energy efficiencies. Nevertheless, the strongly acidic operating conditions necessitate the use of noble metal catalysts, expensive component materials, and membranes based on fluorinated polymers, which together contribute to high costs, environmental concerns, and limited scalability.^6^ In contrast, AWE represents a mature and robust technology that benefits from the alkaline environment, which allows a broader selection of less expensive materials, with reduced environmental impact. However, the typical use of a porous diaphragm to separate the anode and cathode results in increased gas crossover and mixing, placing challenges when operating under intermittent power supply conditions, commonly associated with renewable energy sources.^7,8^ More recently, research efforts have increasingly focused on anion exchange membrane water electrolyzers (AEMWEs). These systems aim to combine the advantages of an alkaline chemical environment with the presence of a nonporous anion exchange membrane, reducing the mixing of gaseous hydrogen and oxygen.^9,10^ Unfortunately, quaternary ammonium salts, which are commonly used as cationic groups in AEMs, are prone to multiple degradation mechanisms under alkaline conditions, particularly in the presence of supporting electrolytes such as KOH 1 M.^10,11^ This compromises the long-term durability of the membranes and represents a major barrier to their large-scale deployment. Nevertheless, considerable research efforts are underway to develop more chemically stable alternatives.^12–14^ Even more recently, a new class of polymeric membranes for water electrolysis in alkaline media has emerged: ion-solvating membranes (ISMs). Unlike AEMs, these membranes do not rely on cationic groups but instead contain acidic functionalities that deprotonate under alkaline conditions, thereby acquiring a negative charge. The resulting polar environment along the polymer backbone enables the transport of KOH and water through the membrane matrix.^10,15^ In order to achieve adequate ionic conductivity, ISMs require a supporting electrolyte with a concentration similar to that employed in conventional AWE systems (e.g., 25 wt % or more).^15^ Moreover, the nonporous structure of ISMs helps to minimize gas crossover, while the absence of cationic groups contribute to improved chemical durability.^15^ To date, polybenzimidazoles (PBIs) have been the most extensively studied polymers for ISMs, used either alone, in blends, or with structural modifications aimed at enhancing their performance.^10,15–18^ The benzimidazole moiety features an acid nitrogen atom with a p*K*_a_ of approximately 13, allowing for the delocalization of a negative charge between the two nitrogen atoms under strongly alkaline conditions.^16^ This characteristic imparts high ionic conductivity to PBI-based membranes, with reported values exceeding 100 mS cm^–1^.^17^ Despite the absence of cationic groups, the presence of heteroatoms in the PBI backbone makes it susceptible to degradation mechanisms under strongly alkaline conditions.^18^ For this reason, it is essential to expand the investigation toward alternative polymer backbones that are free of heteroatoms, focusing on convenient, cost-effective, and scalable synthetic strategies.^10^ In this framework, Hu et al. prepared a highly stable poly­(oxindole biphenylene) ISM that reached 1.9 V at a current density of 2.0 A cm^–2^ in an electrolyzer cell test.^19^ Caielli et al. have used the poly­(biphenylpiperidines) as a polymeric scaffold enriched with acidic moieties to enhance its ionic conduction.^20^ Serhiichuk et al. and Xia et al., in various studies, explored the introduction of tetrazole or carboxylate functionalities into polymer backbones based on polystyrene, poly­(arylene alkylene)­s, and poly­(vinyl alcohol) to promote ionic conduction.^21–24^ In contrast to previously reported non-PBI ISMs, the present one employs a novel copolymer obtained from cheap and widely available commercial monomers (styrene and acrylic acid) that inherently provides both hydrophilic domains and ion-coordinating carboxylic functionalities. The choice of these widely available and low-cost monomers is driven by the goal of developing a cost-effective ISM that can be manufactured on a large scale. Moreover, rather than forming free-standing films, the ionomer is incorporated into a porous polypropylene support to generate a composite ISM with significantly enhanced dimensional stability. This combination of a simple polymer backbone, the absence of any chemical modification steps, and the use of an inexpensive and scalable support architecture represents a notable shift toward the practical implementation of ISMs in alkaline devices. The resulting material integrates the ionic conductivity provided by the negatively charged carboxylate groups formed at the operating pH with the chemical stability of a heteroatom-free polymer backbone and the robust mechanical performance of the polypropylene scaffold, distinguishing it from all previously reported ISM architectures. The composite membrane exhibits, in fact, both a high ion exchange capacity (IEC) and good mechanical strength. Its electrochemical properties were investigated by through-plane conductivity measurements and by testing in a single-cell electrolyzer. The polymer’s chemical resilience was assessed by ^1^H NMR following an aging treatment in KOH 4 M at 70 °C for 720 h. In addition, long-term electrolysis stability was examined through a 150 h discontinuous test, during which three polarization curves were collected. Since no evidence of chemical or functional degradation was observed under any of the testing conditions explored, the potential of this new class of materials for application in ISMs is apparent.

## 2 Materials and Methods

### 2.1 Materials

Styrene (stabilized for synthesis), acrylic acid (stabilized with hydroquinone monomethyl ether for synthesis), azobis­(isobutyronitrile) AIBN (98%), ethyl alcohol (95%), dioxane (99%), potassium hydroxide (85%), PTFE (60 wt % PTFE dispersion in H_2_O) were purchased from Merck-Life Science and used without further purification. Pt/C powder (40 wt % platinum on carbon) were purchased from Thermo Fisher Scientific. Nafion dispersion D520CS were purchased from Ion Power. Carbon cloth porous transport layer (0.410 mm thickness) was purchased from Xiamen Zopin New Material Limited (China) while Nickel foam (1.5 mm) was obtained from Xiamen Tmax Battery Equipment Limited (China).

### 2.2 Polymer Synthesis

In a 200 mL glass reactor equipped with a magnetic stirrer, 9.47 mL of styrene (100 mmol), 5.07 mL of acrylic acid (67 mmol), 250 mg of AIBN (1.5 mmol), were mixed. The free-radical polymerization was carried out at 70 °C under open-air conditions. The mixture was maintained under magnetic stirring, leading to the formation of a solid material within a few minutes. For purification, the poly­(styrene-*co*-acrylic acid) copolymer was dissolved in a 3:2 (v/v) dioxane/ethanol mixture and subsequently precipitated in water; the process was repeated twice. The purified copolymers were dried in an oven at 45 °C for 2 days. Purity and composition were checked by ^1^H NMR spectroscopy.

### 2.3 Membrane Fabrication

Celgard 2400 (Celgard, Charlotte, CA, USA) sheets were impregnated with a 35% w/v solution of Poly­(styrene-*co*-acrylic acid) in a 3:2 (v/v) dioxane/ethanol mixture and dried overnight.

### 2.4 Characterization Methods

#### 2.4.1 ^1^H NMR

Spectra were recorded at 25 °C with a Bruker Avance Ultrashield 400 spectrometer dissolving 5 mg of polymer, previously dried under vacuum, in 600 mg of DMSO-*d*_6_, δ [(CD_3_)­(CHD_2_)­CO] = 2.05. The molar ratio between styrenic units, denoted as **p** in Scheme 1 and Figure 1, and acrylic units, denoted as **q** in Scheme 1 and Figure 1, (i.e., the degree of functionalization) was determined from the ^1^H NMR spectrum by integrating the aromatic protons assigned to signal **b** (Figure 1) and the aliphatic protons assigned to signal **a** (Figure 1); using the following eqs (eqs 1–3)­

1 $$p_{\left({\%}_{mol}\right)}=\frac{\int b/5}{\left(\int a_{eff}/3\right)+\left(\int b/5\right)}\cdot 100$$

2 $$q_{\left({\%}_{mol}\right)}=\frac{\int a_{eff}/3}{\left(\int a_{eff}/3\right)+\left(\int b/5\right)}\cdot 100$$

where ∫*a*_eff_ is defined as

3 $$\int a_{eff}=\int a-\left(\int b\times \frac{3}{5}\right)-\int DMSO$$

**Figure 1:**
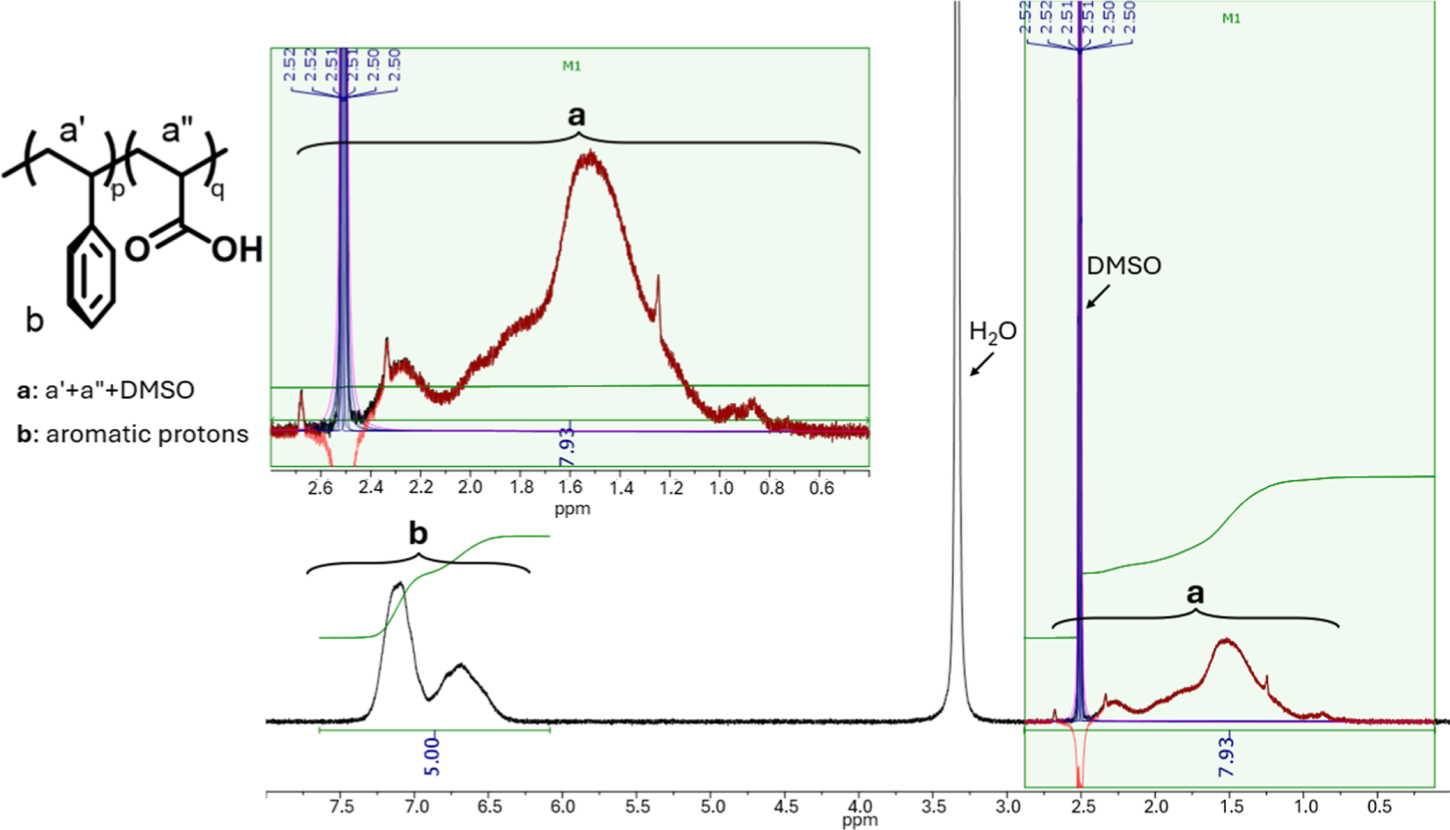
^1^H NMR spectrum of poly­(styrene-*co*-acrylic acid) copolymer.

**Scheme 1:**
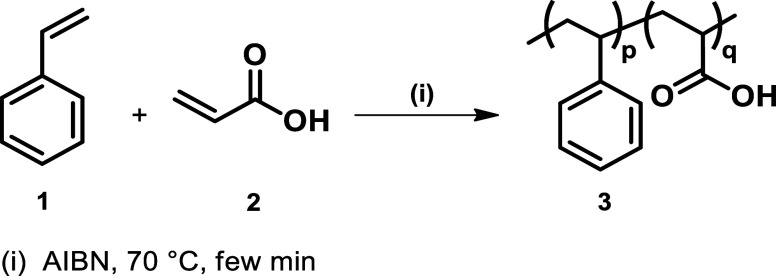
Synthesis of poly­(styrene-*co*-Acrylic Acid) Copolymer

#### 2.4.2 OH^–^ Exchange

Membranes were immersed in abundant 4 M KOH solution for 8 h to exchange.

#### 2.4.3 Thermogravimetric Analysis and Differential Scanning Calorimetry

Thermogravimetric analysis (TGA) was carried out using a TGA 4000 instrument (PerkinElmer Inc., Shelton, USA) in the temperature range 40–600 °C, with a heating rate of 5 °C min^–1^ under a nitrogen atmosphere (40 mL min^–1^). Differential scanning calorimetry (DSC) measurements were performed on a Nexta DSC200 (Hitachi High-Tech Corporation, Japan) between −35 and 200 °C, applying a heating rate of 10 °C min^–1^ under nitrogen flow.

#### 2.4.4 Scanning Electron Microscopy

Scanning electron microscopy (SEM) observations were performed on a Nova NanoSEM 450 field-emission instrument (FEI–Thermo Fisher Scientific, Waltham, MA, USA) operated at 1–5 kV. Both the Everhart–Thornley (ETD) and Through-Lens (TLD) detectors were employed for high-magnification imaging. Prior to analysis, the samples were coated with a thin Au–Pd layer using a Desk V sputter coater (Denton Vacuum, USA).

#### 2.4.5 Stress–Strain Mechanical Tests

Mechanical tests were performed at room temperature using a Shimadzu AGS-X (Shimadzu Corporation, Kyoto, Japan). Stress–strain curves were recorded for the support and the membranes in dry H^+^ form. Samples were cut into rectangular strips (70 mm × 7 mm) and elongated until failure at a constant crosshead speed of 3 × *L*_0_ mm min^–1^, with *L*_0_ corresponding to an initial gauge length of 30 mm. The values of Young’s modulus were determined through independent mechanical tests using the same crosshead speed.

#### 2.4.6 Ion Exchange Capacity

The Ion exchange capacity (IEC), expressed as millimoles of acidic groups per gram of membrane (mmol g^–1^), was determined by correlating the degree of copolymer functionalization with the amount of copolymer adsorbed onto the support, using the following eq (eq 4)­

4 $$IEC\left(mmol\;g^{-1}\right)=\frac{\left(w_{ads}\right)}{x_{a}\cdot M_{a}+x_{b}\cdot M_{b}}\cdot x_{a}\cdot 1000$$

were: *w*_ads_ = mass of copolymer adsorbed per gram of membrane (g/g); *x*_*a*_ = molar fraction of acrylic acid units in the copolymer chain; *x*_*b*_ = molar fraction of styrenic units in the copolymer chain; *M*_*a*_ = Molar mass of acrylic acid (g/mol); *M*_*b*_ = Molar mass of styrene (g/mol); *W*_ads_ was assessed by weighing the support before and after the coating process; *x*_*a*_ and *x*_*b*_ was confirmed through integration of the ^1^H NMR diagnostic peaks, as reported in Section 2.4.1.

#### 2.4.7 Electrolyte Uptake

Electrolyte uptake (EU) is defined as the combined uptake of water and KOH; it was quantified through two independent experimental approaches. In the first one, dried membrane pieces were weighed (*m*_dry_) and individually immersed at room temperature in either deionized water or KOH solutions of varying concentrations (2, 4, and 6 M) for 8 h. After the exposure time, the liquid excess was gently removed from the surface and the membranes were reweighed to determine the swollen mass (*m*_wet_). In the second approach, a similar procedure was followed, but all membranes were immersed in KOH 4 M and treated at different temperatures (25 °C, 40 and 70 °C) for 8 h. Electrolyte uptake was calculated using the following eq (eq 5)­

5 $$EU\left(\%\right)=\frac{m_{wet}-m_{dry}}{m_{dry}}\times 100$$

#### 2.4.8 Swelling Ratio

Swelling ratio (SR) was evaluated using two independent experimental approaches, both based on measuring membrane thickness before and after equilibration in alkaline media. Square membrane specimens (0.5 cm × 0.5 cm) were used in both cases. In the first set of experiments, the dry thickness (*l*_dry_) of each sample was recorded, followed by immersion in deionized water or KOH solutions at concentrations of 2, 4, and 6 M for 8 h at room temperature. After equilibration, excess surface liquid was gently removed, and the swollen thickness (*l*_wet_) was measured. In the second set of experiments, samples were immersed in a KOH 4 M solution and equilibrated for 8 h at different temperatures (25 °C, 40 °C, and 70 °C). As before, *l*_dry_ was measured before immersion, and *l*_wet_ was recorded after removing surface liquid. SR was then calculated using the following eq (eq 6)­

6 $$SR\left(\%\right)=\frac{l_{wet}-l_{dry}}{l_{dry}}\times 100$$

#### 2.4.9 Aging Tests

Poly­(styrene-*co*-acrylic acid) samples were soak in 4 M KOH at 70 °C for 720 h. After the test time, the KOH solution was removed, and the polymer samples were immersed in water. A concentrated HCl solution (37 wt %) was then added dropwise until a strongly acidic pH (≈ 1) was reached. The acidic water was subsequently removed, and the polymer was thoroughly washed with fresh water through multiple rinsing and centrifugation cycles, until the washing water reached approximately pH = 6. Eventually, the polymeric sample was dried under vacuum (≈50 mbar) at 50 °C and ^1^H NMR spectrum was recorded and analyzed as reported in Section 2.4.1.

### 2.5 Through-Plane Conductivity

Membrane samples (4 × 4 cm^2^, 60 μm thickness) were cut and soaked overnight in an excess of 4 M KOH solution to allow complete ion exchange. A symmetrical WE cell of 5 cm^2^ active area was assembled using Ni foam (300 μm) electrodes and 300 μm PTFE gaskets, and one membrane with 50 μm PTFE gaskets (20% membrane compression ratio). Five ml min^–1^ of 4 M KOH electrolyte were recirculated at both electrodes. Cell resistance was measured with Electrochemical Impedance Spectroscopy (EIS) (100 mA cm^2^ DC, 20 mA AC, 100 kHz^–1^ Hz freq range) with a Biologic SP-200 each 5 min. A constant current of 100 mA cm^2^ was applied to the cell in-between EIS measurements to keep membrane decarbonated. Initial temperature was set to 30 °C and raised by 10 °C each 45 min up to 70 °C. To evaluate and exclude the contribution of cell parallel resistance, the procedure was repeated with two overlapped membranes, keeping constant membrane compression ratio at 20% with two 50 μm PTFE gaskets. Membrane conductivity was calculated with the following formula (eq 7)­

7 $$\sigma \left[mS\;{cm}^{-1}\right]={\left(\frac{qA}{s}\right)}^{-1}$$

where *q* is the slope of the line obtained by the linear fit of cell resistance/number of membranes lines expressed in *m*Ω *n*_memb_^–1^, *A* is the cell area in cm^2^ (5), *s* is the thickness of one membrane expressed in cm (0.006).

#### 2.5.1 Electrolyzer Cell Tests

Electrolysis experiments were carried out in a single-cell electrolyzer (active area 5 cm^2^, H5, Antares Electrolysis S.r.l., Italy). The anode was prepared by dispersing the catalyst powder (Ni_1–*x*_Co_*x*_/NiCoO_*x*_, synthesized as described by Malaj et al.^25^) in ethanol, followed by the addition of a PTFE aqueous dispersion (60 wt % in H_2_O) in an amount corresponding to 10 wt % of the catalyst. The resulting slurry was manually applied onto a Ni foam support (5 cm^2^) and subsequently dried overnight at 30 °C, giving a catalyst loading of 25 mg cm^–2^. The cathode was obtained by mixing commercial Pt/C with a 5 wt/v % Nafion dispersion, added to reach 10 wt % Nafion relative to the solid content. The ink was deposited on 5 cm^2^ of carbon cloth and dried overnight at 50 °C, resulting in a Pt loading of 1 mg cm^–2^. For Membrane Electrode Assembly (MEA) preparation, both electrodes were pretreated in 4 M KOH for 30 min and then assembled with the membrane. Polarization curves were recorded by applying stepwise current densities while monitoring the cell voltage using a Hioki 3560 AC mW HiTester (Hioki Corporation, Japan). The electrolyte (4 M KOH) was continuously circulated with a peristaltic pump at 20 mL min^–1^. Durability tests were performed under the same flow rate at 70 ± 2 °C using 4 M KOH solution. The experimental setup was configured to detect any current flow, including possible leakage currents, even when the power supply was not actively delivering current.

## 3 Results and Discussion

Poly­(styrene-*co*-acrylic acid) copolymers exhibit two key features that make them particularly suitable for use in ISMs: the absence of heteroatoms along the polymer backbone, and the presence of acidic functional groups. Under alkaline conditions, these carboxylic groups can be deprotonated, acquiring a negative charge that facilitates the transport of water and hydroxide ions, particularly in concentrated alkaline solutions. The reaction between styrene **1** and acrylic acid **2** was conducted in bulk using azobis­(isobutyronitrile) (AIBN) at 70 °C (Scheme 1).

The monomer feed ratio was optimized by qualitatively evaluating the behavior of the resulting copolymer in 4 M KOH at 80 °C. The aim was to incorporate the highest possible density of carboxylic groups, which are mainly responsible for ionic conductivity, without inducing solvation or excessive swelling of the polymer matrix. A styrene-to-acrylic acid molar ratio of 3:2 was found to offer the most favorable balance between functionality and structural stability (see Supporting Information and Materials and Methods section). The ^1^H NMR spectrum in DMSO-*d*_6_ of the poly­(styrene-*co*-acrylic acid) copolymer **3** obtained from the reaction in Scheme 1 is shown in Figure 1.

In the spectrum of **3** (Figure 1) it is possible to distinguish two separate spin systems: the first corresponds to the aromatic protons of the styrenic units, labeled as **b** in Figure 1, while the second includes the aliphatic protons from both styrenic and acrylic units, labeled as **a** in Figure 1. The calculation of the stoichiometric ratio of the repeating units, performed according to eqs 1 and 2 reported in Section 2.4.1, revealed a composition of 71% styrenic units and 29% acrylic units within the copolymer chain; resulting in a lower fraction of carboxylic groups in the polymer backbone compared to the monomer feed ratio used during synthesis. The resulting poly­(styrene-*co*-acrylic acid) **3** is a transparent, stiff, and brittle solid, which swells and becomes opalescent and malleable upon immersion in a 4 M KOH solution. Interestingly, the copolymer remains insoluble in water, even at 100 °C, when it is fully protonated (H^+^ form). Immersion in KOH solutions from 0.1 to 1.8 M deprotonates the carboxylic groups, creating negatively charged sites that enable the polymer to dissolve at room temperature. However, when the KOH concentration exceeds approximately 2 M, the increased ionic strength screens electrostatic repulsions and promotes salting-out, thereby preventing polymer dissolution, an effect commonly observed in polyelectrolytes.^26,27^ Moreover, once equilibrated in KOH, the polymer becomes soluble even upon transfer to neutral water. This indicates that the membrane can stably operate only in the presence of supporting electrolytes with KOH concentrations above 2 M. However, these conditions are commonly employed in ISM applications. In order to obtain a homogeneous and mechanically robust membrane, the copolymer was supported onto a commercial microporous monolayer membrane made of polypropylene (PP) with 41% porosity and 25 μm thickness (Celgard 2400).^28^ The polypropylene support enables the fabrication of a uniform and mechanically robust, composite ISM (Figure 2d).

**Figure 2:**
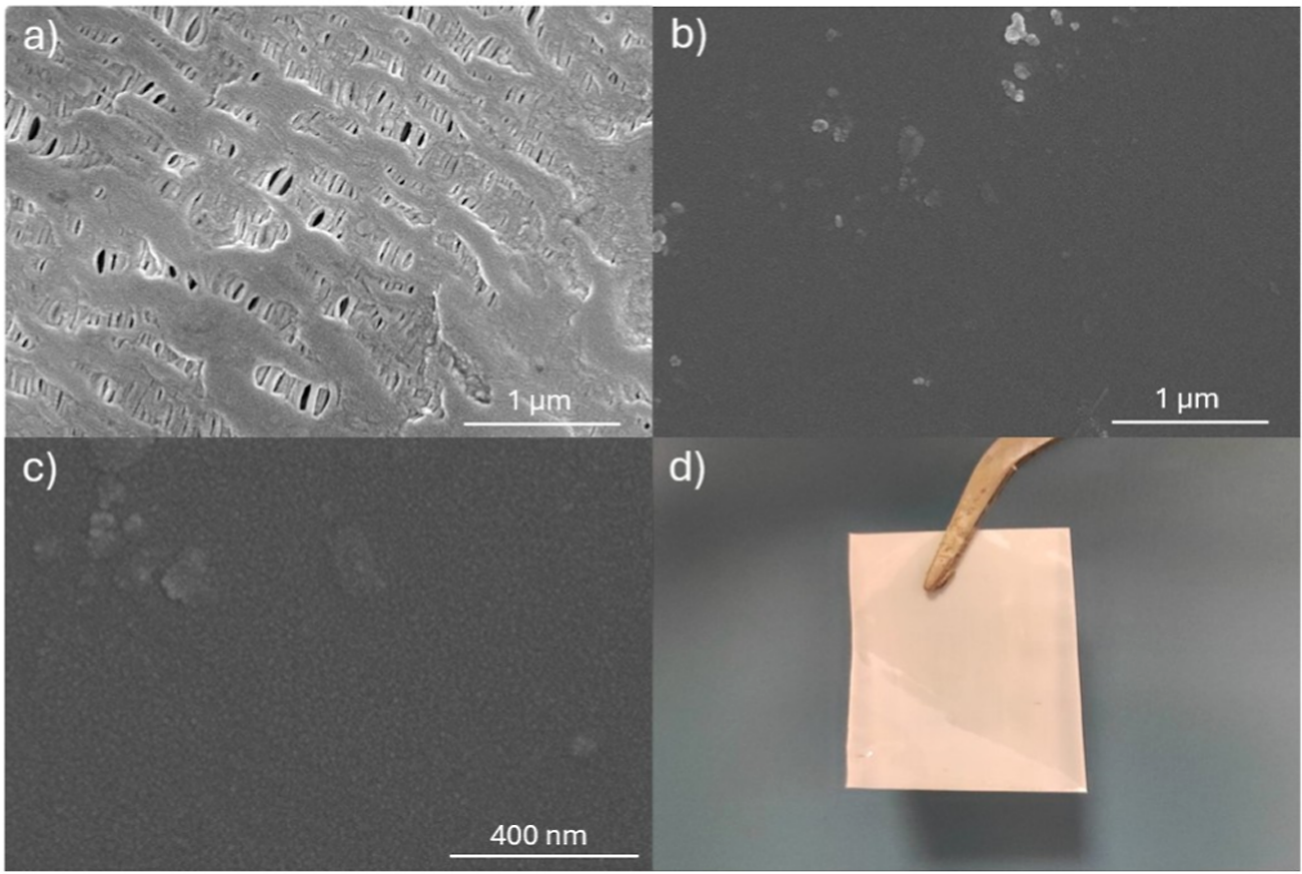
SEM images of: (a) pristine Celgard 2400 microporous commercial film (100,000× magnification) adapted from Lentini et al., Electrochimica Acta, 2025, under CC BY 4.0 license,^14^ (b) DLAAS membrane (100,000× magnification), (c) DLAAS membrane (300,000× magnification) and (d) Optical picture of the obtained DLAAS membrane (dry, H^+^ form).

Comparison of the SEM images of the bare support, reproduced from Lentini et al.^14^ (licensed under CC BY 4.0) (Figure 2a) and the resulting composite membrane, hereafter referred to as DLAAS (Figure 2b,c), confirms that the coating process led to complete and uniform coverage. The cross-sectional SEM images of the membranes (Figure S1), also reveal complete surface coverage with a compact copolymer layer, along with internal penetration of the copolymer into the support. Since the fabrication process was carried out manually, the final thickness of the membranes in their dry, protonated (H^+^) form varied depending on the volume and speed of the casting procedure. This variability mainly stems from the flow behavior of the polymer solution along the support surface during the drying phase. On average, the membranes exhibited a dry thickness of approximately 80 μm. The amount of polymer absorbed on the support was determined by comparing the weight of the Celgard 2400 substrates before and after coating (in the dry, protonated form). For membranes with an average dry thickness of 80 μm, the polymer loading was approximately 11 mg cm^–2^, corresponding to about 89.5% of the total membrane weight.

The TGA profile of the fabricated membrane is shown in Figure 3. The initial mass loss of 8% up to 125 °C is attributed to the evaporation of residual solvents and water, which is consistent with the hydrophilic nature of the charged polymer backbone and its ability to retain water within the membrane matrix. A subsequent weight loss of 6% is observed between approximately 125 and 255 °C, likely corresponding to partial thermal degradation of the poly­(styrene*-co-*acrylic acid), particularly in the acrylic segments.^29^ A final significant decomposition step occurs near 400 °C, which can be ascribed to the breakdown of the polypropylene support,^30^ along with further degradation of the copolymer.

**Figure 3:**
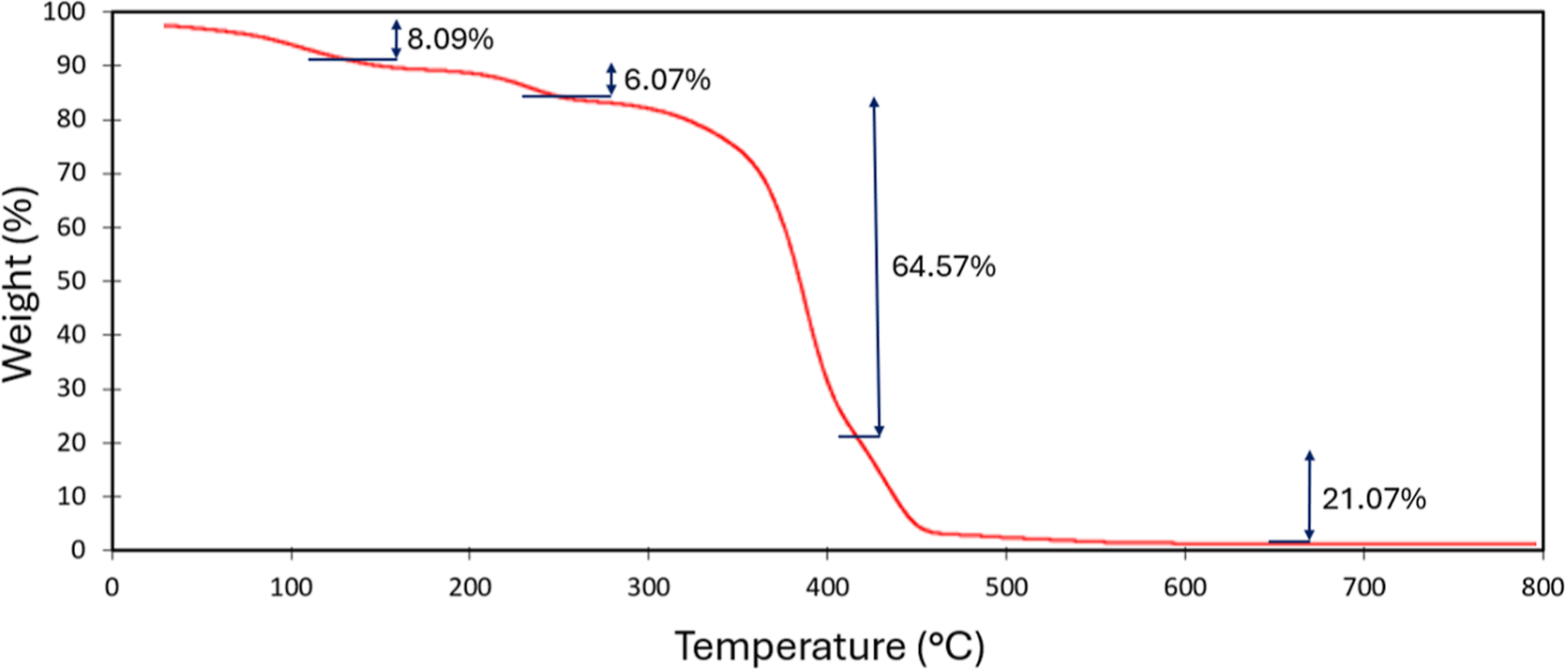
Thermogravimetric analysis of DLAAS membrane.

As shown in Figure 4, the DSC analysis reveals the absence of any distinct thermal transitions associated with the crystallization or melting of the poly­(styrene*-co-*acrylic acid) within the investigated temperature range. In particular, the first heating curve is characterized by a broad signal in the range of 45–85 °C, which is likely due to residual solvent(s) evaporation, and by a wide endothermic peak centered at 164.5 °C attributable to the melting of the PP support.^30^ Crystallization (peak centered at 116.8 °C) and melting (peak centered at 158.6 °C) of the PP support are also evident in the cooling and second heating curves, respectively. It is worth pointing out that TGA and DSC analyses confirm that the DLAAS membrane is thermally stable in the operating range of an ISM.

**Figure 4:**
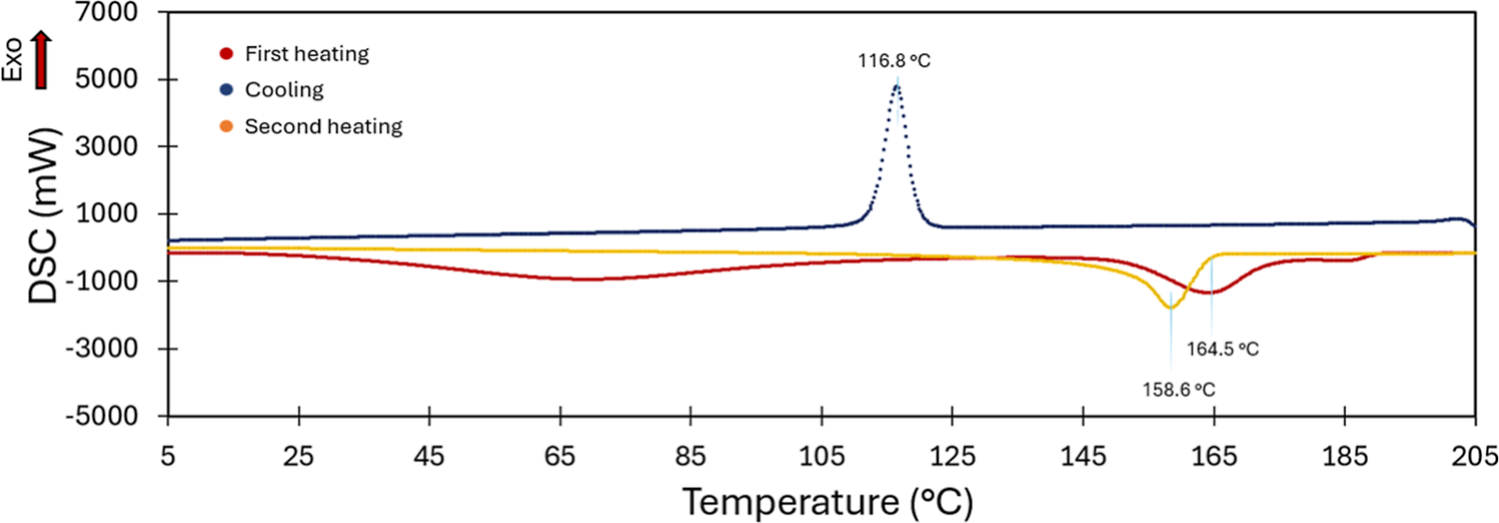
DSC of DLAAS membrane.

The stress–strain curves of the dry membrane are reported in Figure 5; the membrane’s composite design imparts good mechanical properties, with a Young’s modulus of 1496 ± 349 MPa a stress at break of 123 ± 14 MPa and an elongation at break of 65 ± 17%. These values are comparable to those of the bare support and slightly higher than those of the membranes after activation in 4 M KOH (Figures S2, S3 and Table S1), confirming—as expected- that the mechanical properties of the composite membrane are largely governed by the polypropylene substrate. The slight decrease observed after alkaline activation is consistent with the expected plasticizing effect of KOH, which leads to a modest reduction in stiffness and tensile strength without compromising the overall structural integrity of the material. This further supports the conclusion that the polypropylene scaffold provides the dominant contribution to the mechanical behavior of the composite. It is worth noting that poly­(styrene*-co-*acrylic acid), when dry and in its (H^+^) form, is brittle and prone to surface flaking if the membranes are not handled with care (Figure S1).

**Figure 5:**
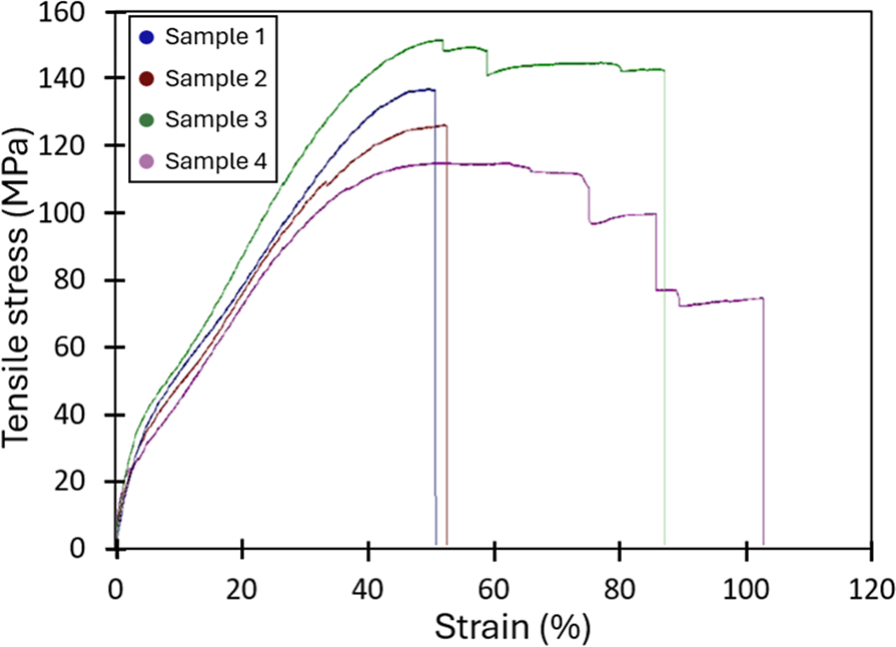
Stress strain curves of DLAAS membrane.

It should be emphasized that the polypropylene substrate exhibits pronounced structural anisotropy (Figure 2a), which translates into direction-dependent strength.^28,30^ As a result, the same anisotropic behavior is observed in the composite membrane. The curves displayed in Figure 5 were recorded along the machine direction, parallel to the oriented fibrils identified in Figure 2a. In contrast, tests conducted in the transverse direction, perpendicular to those fibrils, were not reproducible due to early failure. This pronounced anisotropy does not compromise the mechanical robustness or the handling of the membranes during either fabrication or electrochemical testing. Overall, the results suggest that the membrane exhibits sufficient mechanical integrity to withstand the stresses typically encountered during electrolytic cell operation.

The IEC, expressed in millimoles of carboxylic acid groups per gram of membrane (mmol g^–1^), was calculated by combining the degree of functionalization of the poly­(styrene-*co*-acrylic acid) copolymer (29%) with the average mass of copolymer adsorbed onto the support (approximately 11 mg cm^–2^), as described in the eq 4 reported in Section 2.4.6. Considering that 1 g of membrane contains 0.895 g of copolymer and that the molar fraction of acrylic units in the polymer chain is 0.29, the resulting IEC is 2.72 mmol g^–1^.

EU and SR were monitored using two different approaches: in the first one, the temperature was kept constant (25 °C) while varying the electrolyte concentration (Figure 6a,b); in the second one, the concentration was fixed (4 M) and the temperature was varied (Figure 6c,d), checking that the membrane weight at 25 °C before and after heating remained unchanged. Since no significant dimensional changes were observed in-plane, swelling was evaluated based on variations in membrane thickness. The maximum temperature used during the tests was 70 °C. Above this threshold, surface embrittlement of the membrane was observed and, although limited, partial detachment of the polymer layer cannot be ruled out. As the electrolyte molarity increases, both the EU and the SR initially rise when moving from deionized water to 2 M KOH. At higher concentrations (4 and 6 M), both values decrease but remain higher than those measured in neutral water (Figure 6a,b). When the electrolyte concentration is fixed at 4 M, both the EU and SR exhibit similar trends, reaching a maximum at 40 °C, with only slight variations compared to the values observed at 25 and 70 °C (Figure 6c,d). Since DSC analysis (Figure 4) did not reveal any thermal transitions or softening phenomena, indicating that neither the copolymer nor the polypropylene support undergo structural or mechanical changes within this temperature range, the maximum observed at 40 °C cannot be attributed to thermally induced transitions but is instead ascribed to a balance between the increased molecular mobility of the electrolyte and osmotic dehydration effects. A similar trend has been reported for other ISMs and is generally attributed to two main phenomena: Donnan exclusion and osmotic dehydration.^20,21^ The former is due to the presence of fixed negative charges within the membrane and their electrostatic influence, which restricts the uptake of K^+^ and OH^–^ ions despite their external concentration increases. The latter is driven by the decreasing water activity at higher KOH molarity, which induces water efflux from the membrane due to the osmotic gradient. Together, these effects result in a progressive decrease in both EU and SR with increasing KOH concentration, a behavior previously observed for Nafion in concentrated saline media.^31^ Notably, these phenomena appear to be only slightly affected by temperature under constant molarity conditions. The insertion into a PP porous matrix allows to obtain good EU and SR values that, together with the very good mechanical performances, constitute an optimal condition for use in large-scale electrolytic cells where size retention is essential during assembly and operation.^10^ To achieve an appropriate balance between the ionic conductivity of the electrolyte, the electrolyte uptake of the membrane, and to remain within the operating range traditionally employed in alkaline water electrolyzers as well as in other studies on ISMs, the electrochemical tests were carried out in 4 M KOH solutions.

**Figure 6:**
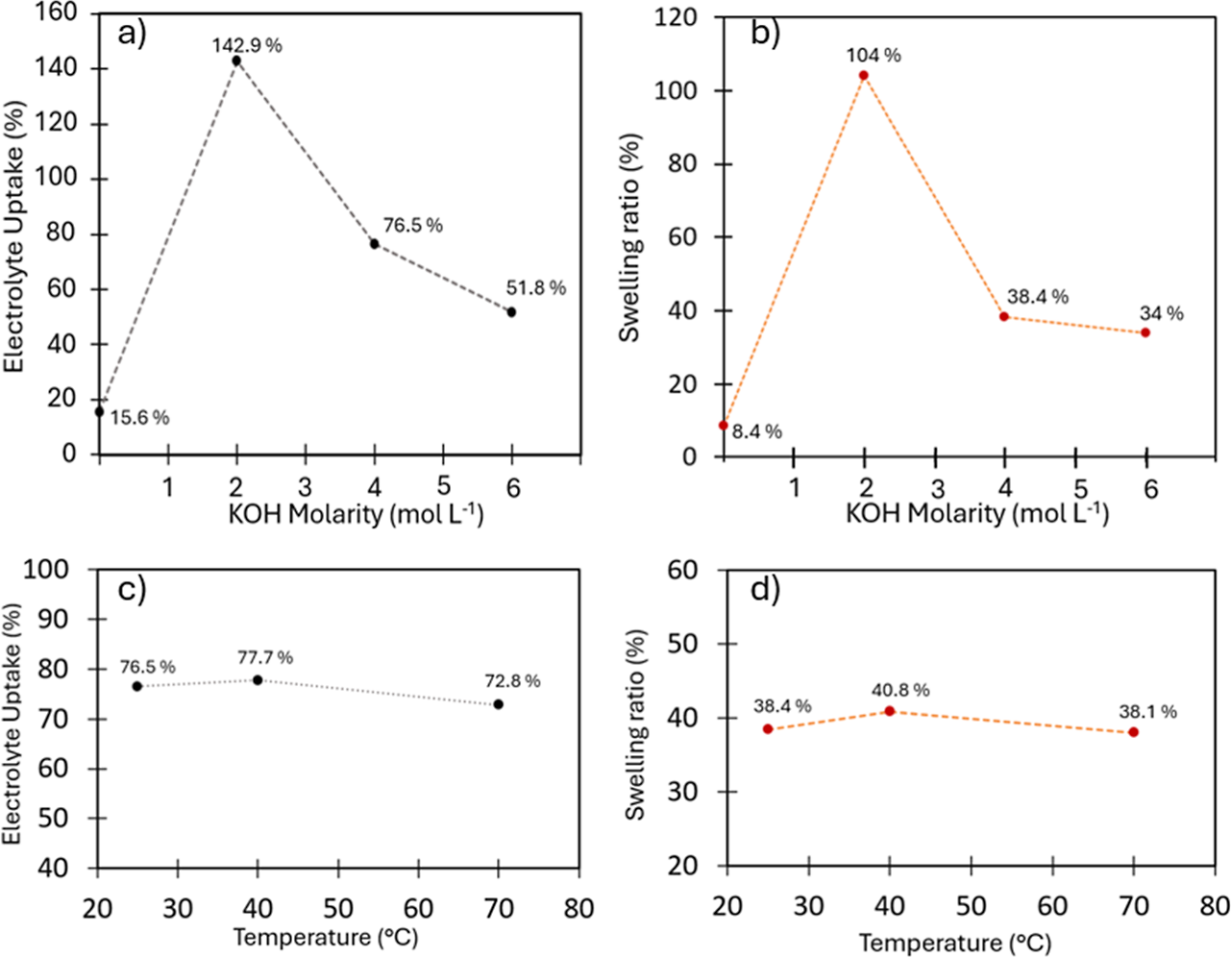
(a) EU vs KOH molarity, (b) SR vs KOH molarity, (c) EU vs temperature, (d) SR vs temperature.

The through-plane conductivity was measured by stacking multiple membrane layers until reaching a total thickness of 120 μm, using 4 M KOH at 70 °C as the supporting electrolyte. Conductivity values were calculated from the slope of the linear fit of resistance versus thickness (Figure S4). The corresponding resistance and ionic conductivity values as a function of temperature are presented in Figure 7a and summarized in Table S2. From these data, the temperature dependence of hydroxide transport was further analyzed by plotting the natural logarithm of the conductivity (expressed in mS cm^–1^) against the inverse absolute temperature (Arrhenius representation, Figure 7b). The resulting linear trend was used to extract the activation energy for OH^–^ conduction, calculated from the slope of the fit according to the Arrhenius equation. The derived activation energy was found to be 43.0 kJ mol^–1^. Given the lack of Arrhenius-type conductivity analyses for ISMs containing carboxylic acid functionalities and the fundamental differences between our hydroxide/carboxylate system and the ionic environments typically discussed in the literature, this value is reported solely as an experimental parameter describing the temperature dependence of conductivity, without inferring a specific ion transport mechanism. To this last aim, a more detailed mechanistic investigation will be addressed in future studies.

**Figure 7:**
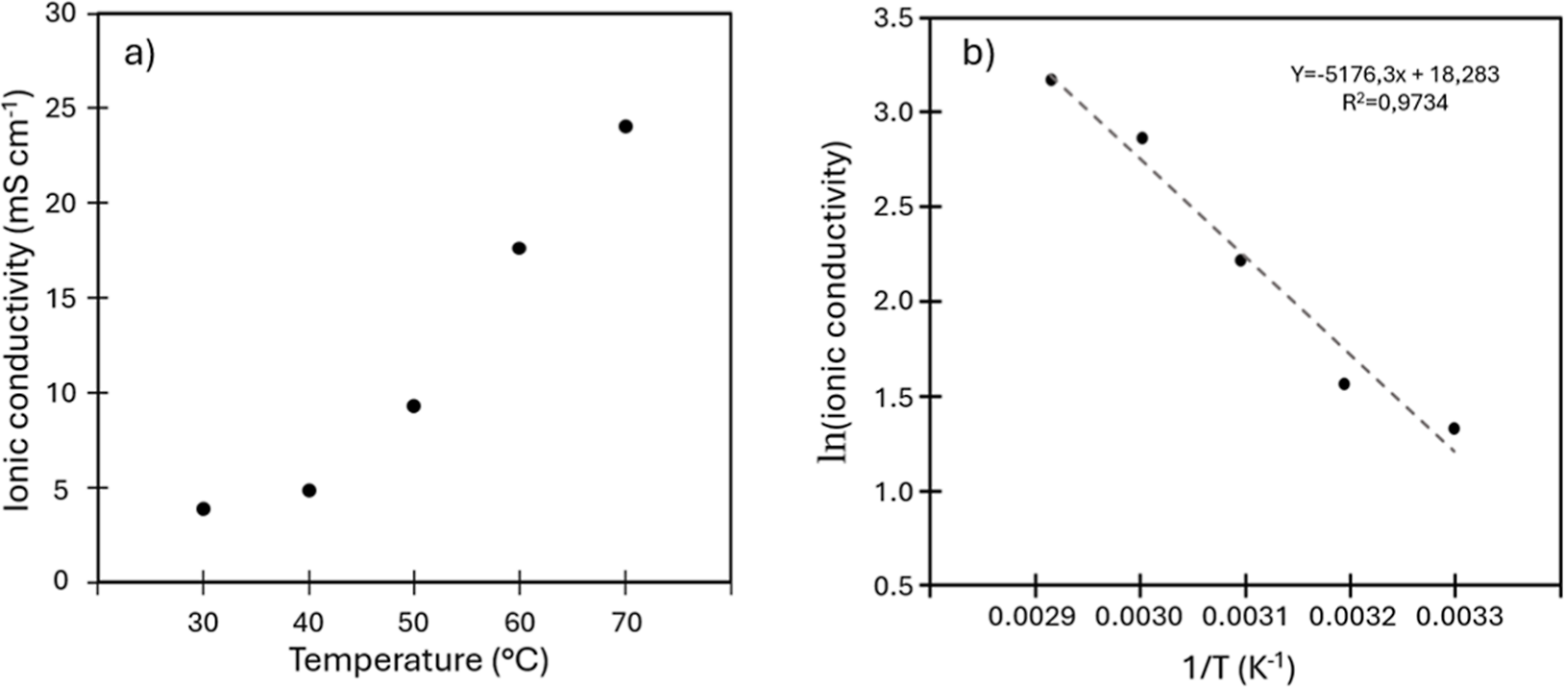
(a) Through-plane ionic conductivity vs temperature (b) Arrhenius plots of DLAAS membrane.

To assess the practical applicability and electrochemical stability of the ISM developed in this work, electrolysis tests were conducted in a single-cell electrolyzer with an active area of 5 cm^2^, operating with 4 M KOH at 70 °C as the supporting electrolyte. Commercial Pt/C was used as the cathode electrocatalyst, while Ni_1–*x*_Co_*x*_/NiCoO_*x*_ nanoparticles, previously investigated in an earlier study, served as the anodic catalyst.^25^ The electrochemical performance was monitored over a total of 150 h following a daily discontinuous operation protocol (Figure 8). Each day, the electrolyzer was operated at a constant current density of 0.4 A cm^–2^ for 8 to 10 h, with gradual start-up and shut-down phases at 0.2 A cm^–2^ lasting 30 min each (not included in the 150 h mentioned above). At the end of each cycle, the system was shut down overnight with no current applied. This procedure was designed to realistically simulate intermittent operating conditions typical of renewable energy-powered systems and to expose the membrane and electrodes to repeated start-up and shut-down stress. It is worth pointing out that these operating conditions were intentionally selected for the purpose of evaluating the intrinsic durability of the membrane: the same anodic and cathodic catalysts used here had previously demonstrated negligible degradation during long-term electrolysis at 0.4 A cm^–2^, ensuring that any performance variations could be attributed to the membrane only rather than to catalyst aging.^25^ It is also worth noting that the maximum current density reached in this study (1.0 A cm^–2^, as shown in the polarization curves) already exceeds the typical operating conditions of conventional alkaline electrolyzers and falls within the range commonly reported in recent AEM and ISM literature. The exploration of higher, industrial-level load conditions has been neglected since it lies beyond the scope of this fundamental study, whose primary aim is to demonstrate the feasibility and stability of this new ISM material under well-controlled laboratory conditions. Polarization curves were recorded at 0, 100, and 150 h across a current density range from 0 to 1 A cm^–2^ to monitor performance over time (Figure 9 and Table S3).

**Figure 8:**
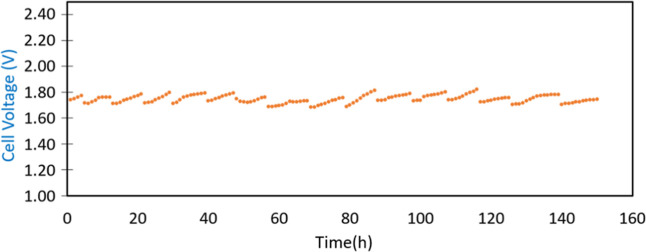
Degradation trend of DLAAS at 70 °C with a constant current density of 0.4 A cm^–2^.

**Figure 9:**
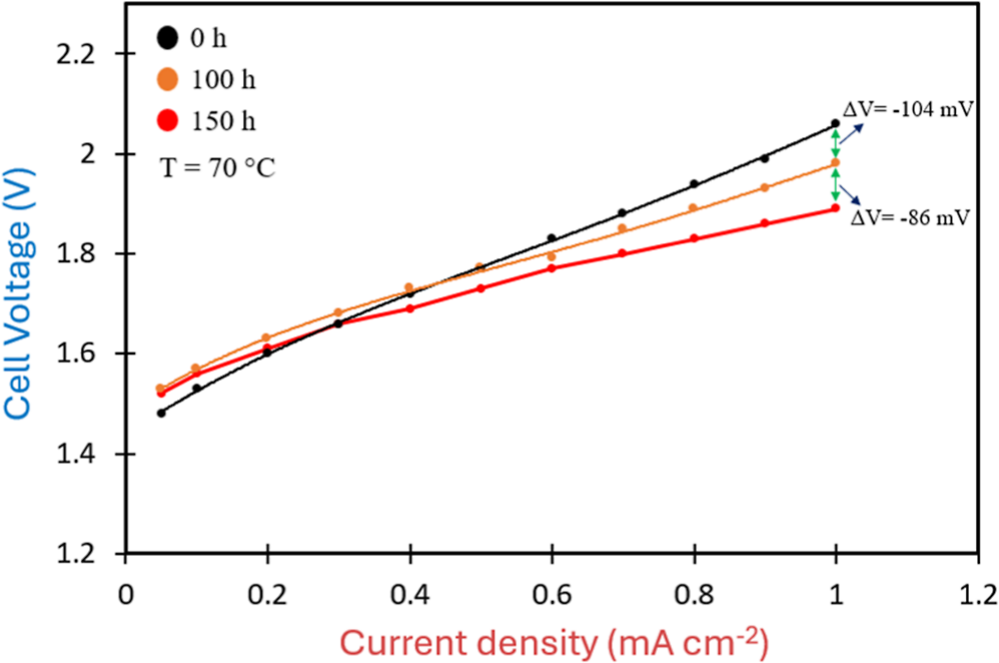
Polarization curves comparison of DLAAS at 0 h (black curve), 100 h (orange curve), and 150 h (red curve).

As can be observed from Figure 8, cell performance exhibited some day-to-day fluctuations; however, the overall voltage range between start-up and shut-down phases remained relatively stable throughout the testing period. As shown in Figure 9 and detailed in Table S3, the cell voltages recorded at current densities below 0.6 A cm^–2^ are comparable across all time points. However, a gradual improvement is observed at higher current densities. Notably, the voltage gap at 1 A cm^–2^ between the initial curve and that recorded after 150 h reaches 190 mV, resulting in a final value of 1.89 V. A similar behavior has been previously reported for the anodic catalyst used here,^25^ where an activation process occurring under operating conditions was observed despite the use of a different membrane and cell configuration. This prior evidence supports the hypothesis that a comparable surface activation may also take place in the present system. However, direct postoperation characterization of the electrodes could not be performed, as the MEA was assembled by mechanical compression: under these conditions, a significant portion of the electrocatalyst layer adheres to the membrane, making recovery without damage extremely difficult and preventing reliable post-mortem analysis. For this reason, the performance enhancement observed here cannot be conclusively attributed to a specific morphological or compositional change. For these reasons, although this test offers useful insights into degradation behavior, it should not be regarded as a conclusive endurance assessment from an industrial perspective, where broader operating conditions and significantly longer testing durations are typically required.

An additional test aiming at monitoring polymer degradation was carried out by immersing the copolymer in a 4 M KOH solution heated to 70 °C for 720 h. After this period, the polymer was placed in strong acidic water, washed until mild acidic pH, dried under vacuum, and subsequently analyzed by ^1^H NMR spectroscopy (details in the experimental Section 2.4.9). A comparison between the NMR spectra of the freshly prepared polymer and the aged sample is shown in Figure 10. As clear, no structural changes or formation of degradation products were observed. This aging protocol was selected to closely mimic the alkaline environment experienced by the membrane during operation, providing a direct and meaningful assessment of the intrinsic chemical stability of the poly­(styrene-*co*-acrylic acid) backbone. The widely documented resistance of the Celgard support^28^ to concentrated alkaline media, allowed us to conclude that this copolymer-focused evaluation was sufficient to determine the chemical robustness of the composite membrane. The fresh bulk polymer’s NMR spectrum also shows signals, confirmed by selective spiking of the analyzed samples, corresponding to residual dioxane and water from the processing steps, appearing at 3.58 and 3.34 ppm, respectively.

**Figure 10:**
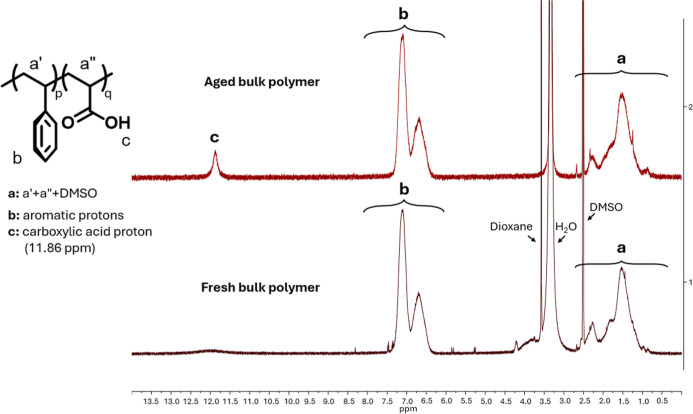
Comparison between the NMR spectra of the freshly prepared (black spectrum) poly­(styrene-*co*-acrylic acid) copolymer and of the aged sample (red spectrum) carried out by immersing the copolymer in a 4 M KOH solution heated to 70 °C for 720 h.

## 4 Perspective and Outlook

Hydrogen produced through water electrolysis powered by renewable energy aims to compete with existing carbon-intensive production methods. To make this possible and to enable hydrogen to play a central role as a low-carbon energy carrier in the energy transition, substantial efforts are required to scale up low-temperature electrolysis technologies. Reaching this objective will also depend on significant cost reductions, streamlined manufacturing processes, and a minimized environmental footprint.^32^ It is within this context that ISMs are positioned, offering the potential to bridge the gap between the broad, cost-effective range of materials typically used in alkaline electrolyzers and the performance advantages of PEM systems: including high efficiency, compatibility with intermittent renewable energy sources and the ability to operate under differential pressure conditions. The goal is to develop membranes that are chemically robust and resistant to alkaline degradation, enabling durable and scalable water electrolysis technologies. With the aim of expanding the range of polymers suitable for this emerging class of membranes, this work explores the use of poly­(styrene-*co*-acrylic acid) copolymer as the ion-conductive component in a composite membrane supported on microporous polypropylene. Although conceptually simple and composed of widely used and low-cost monomers, poly­(styrene-*co*-acrylic acid) offers a valuable combination within the polymer backbone: a chemically stable, heteroatom-free aromatic unit and a carboxylic functional group, capable of deprotonation and electrolyte conduction under alkaline conditions. Since both the copolymer composition and the composite architecture are introduced here for the first time, the present work should be regarded as an exploratory demonstration of feasibility and intrinsic electrochemical functionality of a new class of composite material to be used as ISM. The electrolysis tests performed at moderate current densities were therefore designed to provide clear proof-of-concept evidence while ensuring stable operation and avoiding degradation effects that could complicate data interpretation. One of the goals of this work is also to lay the groundwork for future developments that could further enhance the properties of the proposed material. Potential fields of exploration may include alternative polymerization methods beyond the simple free-radical strategy employed here, as well as adjusting molecular weight and distribution or incorporating a third monomeric unit into the polymer backbone. Although the molecular weight of the present copolymer was not determined, future studies aimed at refining the synthesis route may include a systematic evaluation of this parameter, which could help establish structure–property correlations once the membrane concept has been fully validated. At this stage, however, the feasibility of using poly­(styrene-*co*-acrylic acid) as an ion-solvating matrix was considered the primary objective, and the absence of molecular-weight data does not limit the interpretation of the electrochemical and stability results. Indirect evidence of robustness, namely the unchanged ^1^H NMR profile after alkaline aging and the stable electrochemical performance observed during durability testing, further supports the suitability of this copolymer under the operating conditions employed. Additional efforts could focus on embedding the copolymer into other polymeric matrices to create composite systems, with the goal of further improving the already good mechanical performance described in this study. Another promising direction may involve reducing and standardizing membrane thickness, together with a more comprehensive evaluation of gas transport properties, particularly hydrogen crossover, which was not quantitatively addressed in the present work. Although hydrogen permeability data are only rarely reported in the literature for both ISMs and AEMs, future studies will aim to systematically investigate this parameter to enable a more complete assessment of the advantages offered by this membrane design.

## 5 Conclusion

In this work, a novel ISM was developed by integrating a poly­(styrene-*co*-acrylic acid) copolymer onto a commercially available microporous polypropylene support. The copolymer was synthesized through a straightforward radical polymerization and subsequently casted onto the support, resulting in a membrane with excellent mechanical properties (tensile strength of 65 MPa) and dimensional stability. The membrane exhibited a high IEC of 2.72 mmol g^–1^, limited swelling, and a through-plane ionic conductivity of 24 mS cm^–1^ at 70 °C. Electrochemical validation in a lab-scale electrolyzer confirmed its practical potential, achieving 1.89 V at 1 A cm^–2^ and 70 °C, and maintaining stable performance over 150 h of intermittent operation. Chemical durability of the copolymer was also assessed via ^1^H NMR after immersion in 4 M KOH at 70 °C, confirming the absence of structural degradation. Overall, this study successfully introduces a new candidate in the field of heteroatom-free polymers for ISMs.

## Supplementary Material



## Notes

Cross-sectional fracture-surface SEM images of the composite membrane, stress–strain curves and corresponding mechanical parameters of the pristine commercial PP support and of the membranes activated in KOH, the fitting of cell resistance as a function of membrane thickness, through-plane OH^–^ conductivity values at various temperatures, and a comparison of cell voltage at different current densities for polarization curves acquired at multiple time points are provided in the Supporting Information (PDF)
