# Supplementary material for: Use of Poly(styrene-co-acrylic Acid) in a Composite Ion-Solvating Membrane for Water Electrolysis
Source: ACS Polym Au. 2025 Dec 19;6(1):256–66. doi: 10.1021/acspolymersau.5c00126 (PMC12903460; doi:10.1021/acspolymersau.5c00126)
Supplement: Supplementary file 1 [file lg5c00126_si_001.pdf]

Use of poly(styrene-*co*-acrylic acid) in a composite Ion-Solvating Membrane for Water Electrolysis

Domenico Lentini[a][b], Francesko Malaj[b][c], Alessandro Tampucci[b], Lorenzo Brogi[b], Tommaso Caielli[c], Piercarlo Mustarelli[c], Pierpaolo Minei[d], Massimo Melchiorre[a] Oreste Tarallo[a], Francesco Ruffo[a]*

[a] Department of Chemical Sciences, University of Naples Federico II, Complesso Universitario di Monte S. Angelo, Via Cintia, IT-80126 Naples, Italy

[b] Ne.m.e.sys. s.r.l., via 2 Giugno 8, 50019 Sesto Fiorentino (Fi), Italy

[c] Department of Materials Science, University of Milano-Bicocca, Via Roberto Cozzi 55, Building U5, 20125, Milan, Italy

[d] SPIN-PET, Via R Piaggio, 32, 56025 Pontedera, Italy

Corresponding author: * **Oreste Tarallo: oreste.tarallo@unina.it**

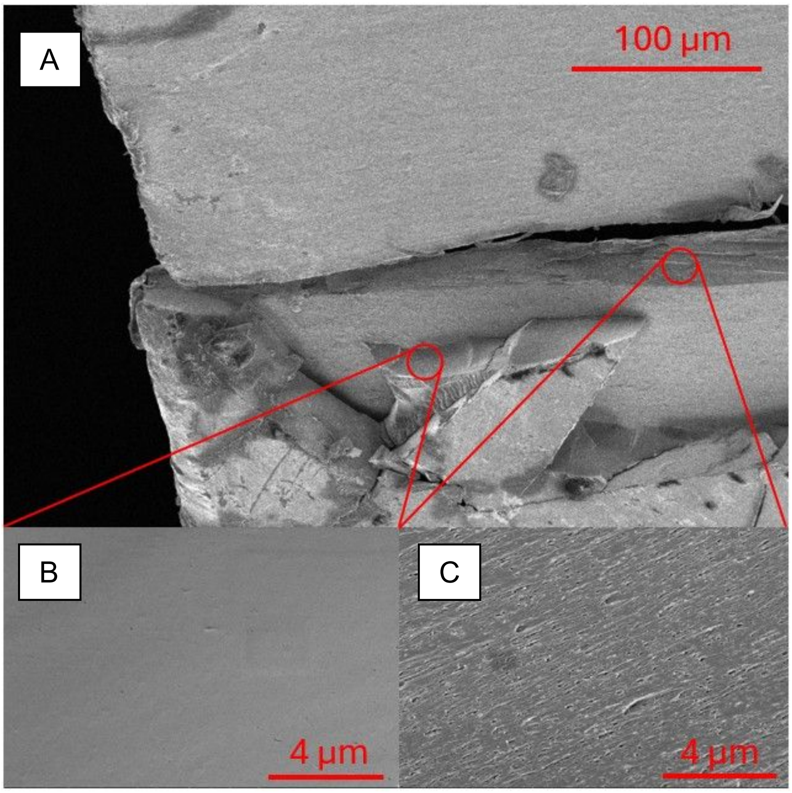

Figure S1: Cross-sectional fracture surface SEM images of the composite membrane ruptured in liquid nitrogen. (A) 1000x magnification. (B, C) 30000x magnification.

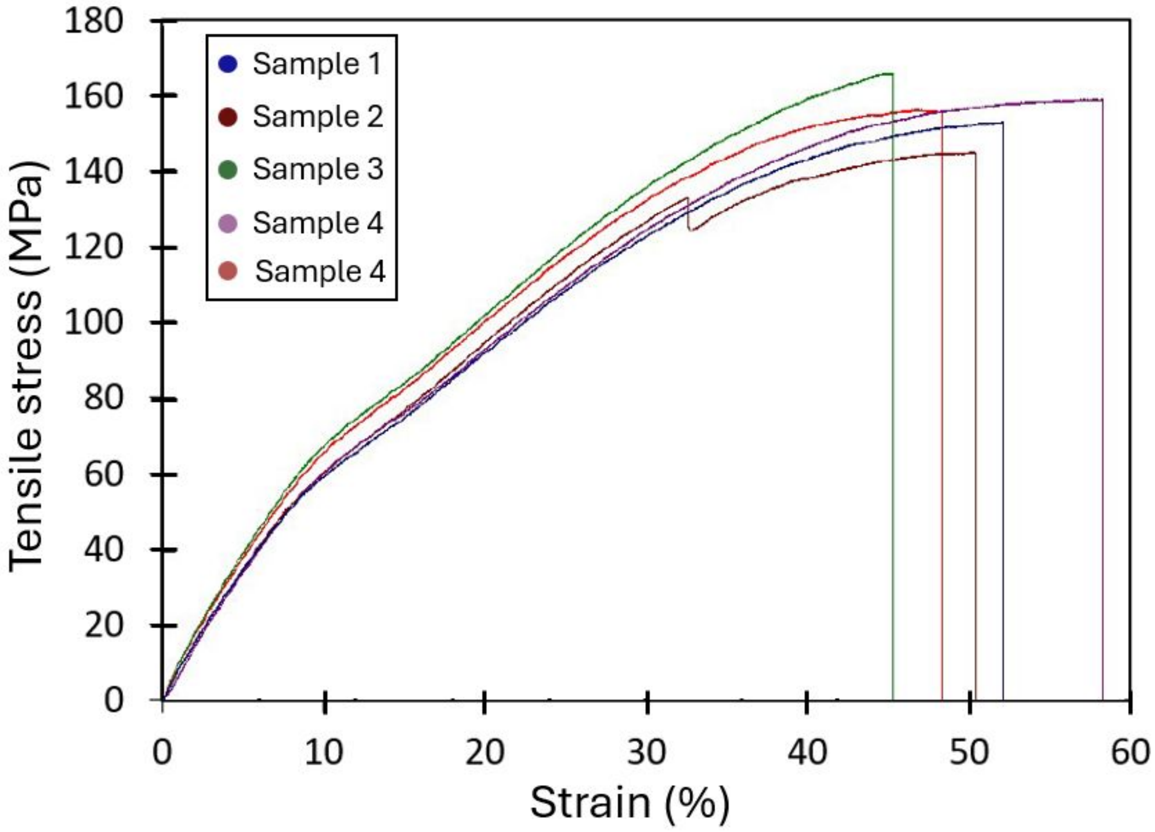

Figure S2: Stress strain curves of pristine commercial PP support.

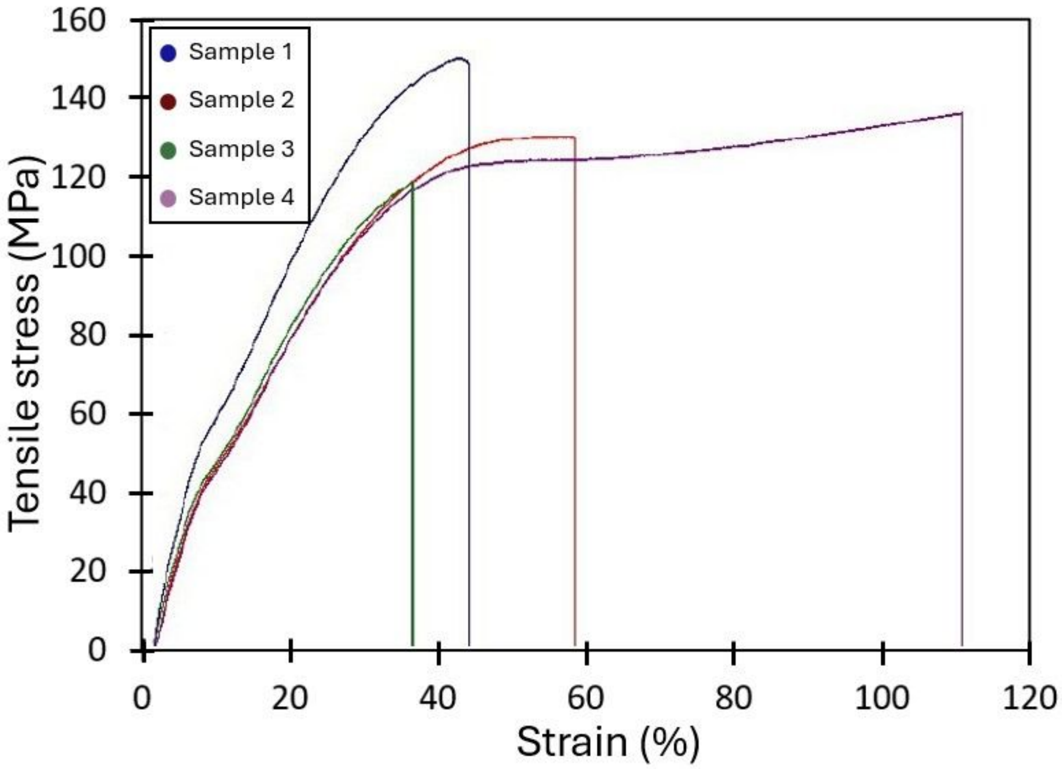

Figure S3: Stress strain curves of the membranes activated in KOH 4M.

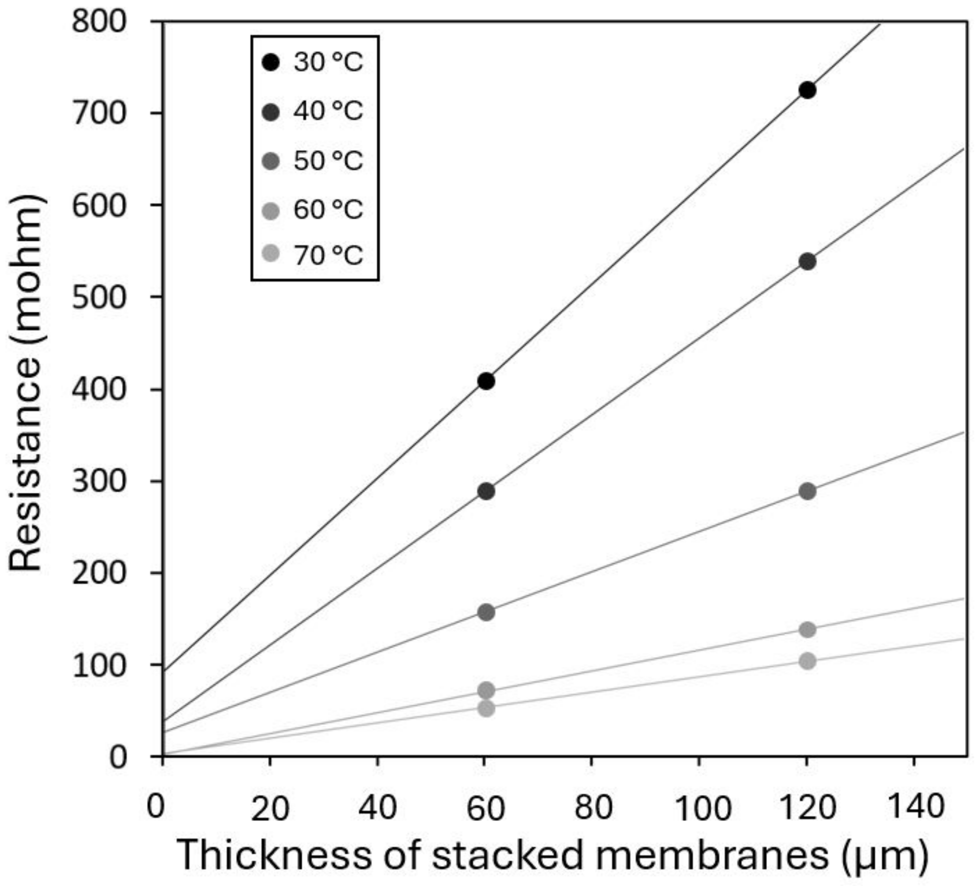

Figure S4: Fitting of cell resistance *vs* thickness

Table S1: Average results for mechanical properties of pristine commercial PP support and membrane after activation in KOH 4M.

**Sample**	**Young modulus [MPa]**	**Elongation at break [%]**	**Stress at break [MPa]**
Support	1099±214	51±5	153±9
4M KOH	849±245	63±34	130±12

Table S2: Through-plane OH^- conductivity values at various temperature

**Temperature [°C]**	**OH^- Conductivity [mS cm^{-1}]**
30	3.8
40	4.8
50	9.2

60	17.6
70	24.0

Table S3: Comparison of cell voltage at various current densities for the polarization curves acquired at different time points

Time [h]	V at 0.2 A cm^{-2}	V at 0.4 A cm^{-2}	V at 0.6 A cm^{-2}	V at 0.8 A cm^{-2}	V at 1 A cm^{-2}
0	1.61	1.72	1.83	1.94	2.1
100	1.63	1.73	1.80	1.89	1.98
150	1.61	1.70	1.77	1.83	1.89
